# Detection and Typing of Highly Pathogenic Porcine Reproductive and Respiratory Syndrome Virus by Multiplex Real-Time RT-PCR

**DOI:** 10.1371/journal.pone.0038251

**Published:** 2012-06-29

**Authors:** Kerstin Wernike, Bernd Hoffmann, Malte Dauber, Elke Lange, Horst Schirrmeier, Martin Beer

**Affiliations:** 1 Institute of Diagnostic Virology, Friedrich-Loeffler-Institut, Greifswald-Insel Riems, Germany; 2 Department of Experimental Animal Facilities and Biorisk Management, Friedrich-Loeffler-Institut, Greifswald-Insel Riems, Germany; Centers for Disease Control and Prevention, United States of America

## Abstract

Porcine reproductive and respiratory syndrome (PRRS) causes economic losses in the pig industry worldwide, and PRRS viruses (PRRSV) are classified into the two distinct genotypes “North American (NA, type 2)” and “European (EU, type 1)”. In 2006, a highly pathogenic NA strain of PRRSV (HP-PRRSV), characterized by high fever as well as high morbidity and mortality, emerged in swine farms in China. Therefore, a real-time reverse transcription polymerase chain reaction (RT-qPCR) assay specific for HP-PRRSV was developed and combined with type 1- and type 2-specific RT-qPCR systems. Furthermore, an internal control, based on a heterologous RNA, was successfully introduced. This final multiplex PRRSV RT-qPCR, detecting and typing PRRSV, had an analytical sensitivity of less than 200 copies per µl for the type 1-assay and 20 copies per µl for the type 2- and HP assays and a high diagnostic sensitivity. A panel of reference strains and field isolates was reliably detected and samples from an animal trial with a Chinese HP-PRRS strain were used for test validation. The new multiplex PRRSV RT-qPCR system allows for the first time the highly sensitive detection and rapid differentiation of PRRSV of both genotypes as well as the direct detection of HP-PRRSV.

## Introduction

Porcine reproductive and respiratory syndrome (PRRS), one of the economically most important diseases of swine worldwide, is characterized by reproductive failure in pregnant sows and respiratory disease in piglets [1]. The causative agent is the antigenically, genetically and clinically heterogeneous PRRS virus (PRRSV) [2]–[4], an enveloped positive-strand RNA virus that belongs to the order *Nidovirales*, family *Arteriviridae* [5]. PRRSV isolates are generally classified into genotype 1 (EU, type 1) and genotype 2 (NA, type 2) [6].

In 2006, a highly pathogenic strain of type 2-PRRSV (HP), causing high fever and severe morbidity and mortality in pigs of all ages, emerged in swine farms all over China [7]. HP-PRRSV, characterized by a unique discontinuous deletion of 30 amino acids (aa) in the non-structural protein 2 (Nsp 2) [7], affected a large number of pigs and causes enormous economic losses [8], [9].

Apart from China, HP-PRRSV has already been detected in other Asian countries such as Vietnam, where it caused a serious epidemic [9], [10]. Therefore it is essential to monitor the spread of these highly pathogenic PRRSV strains. For the detection of HP-PRRSV several real-time reverse transcription polymerase chain reaction (RT-qPCR) assays have been developed [8], [11].

In this study a HP-PRRSV specific RT-qPCR assay was developed and combined with systems specific for type 1 and 2. An internal control (IC) using heterologous RNA [12] was successfully included to verify efficient RNA extraction and uninhibited amplification. Furthermore, an animal trial was conducted with a Chinese HP-PRRSV strain in pigs and the multiplex assay was further validated with samples from the experimental infection.

## Materials and Methods

### Viruses and Diagnostic Samples

The PRRSV strains used in this study (Table 1) were provided by the virus collection of the Friedrich-Loeffler-Institut, Isle of Riems, Germany. The Eastern European isolate “Lena” [13] was kindly provided by U. Karniychuk (Ghent University, Belgium).

**Table 1:** Virus strains used in this study.

Strain	Multiplex PRRSV real-time RT-PCR			ADIAVET PRRS EU/NA	
	PRRSV type 2	HP-PRRSV	PRRSV type 1	PRRSV type 2	PRRSV type1
	Cq	Cq	Cq	Cq	Cq
I 10 (Intervet)	>40	>40	22.84	>40	28.10
Cobbelsdorf	>40	>40	24.66	>40	28.62
Stendal V852	>40	>40	23.22	>40	27.00
Stendal V953	>40	>40	25.27	>40	26.42
Stendal V1904	>40	>40	24.46	>40	27.72
Stendal V1952/97	>40	>40	32.73	>40	28.06
Stendal V1445/99	23.63	>40	>40	26.16	>40
USA, VD28775/2	23.15	>40	>40	24.87	>40
USA, VD29949/17	24.63	>40	>40	27.80	>40
China	23.90	23.95	>40	26.79	>40
USA 2	24.01	>40	>40	26.23	>40
USA 18	20.86	>40	>40	25.68	>40
Lena	>40	>40	25.72	>40	26.04

### RNA Extraction

RNA was extracted with the QIAamp® Viral RNA Mini Kit (Qiagen GmbH, Hilden, Germany) according to the manufacturer’s recommendations, modified by addition of an internal control RNA (IC2) as described by Hoffmann et al. [12], and finally eluted in 50 µl kit elution buffer. RNA from diagnostic serum samples was extracted with the MagNA Pure LC Total Nucleic Acid Isolation Kit for automated extraction (Roche Diagnostics Deutschland GmbH, Mannheim, Germany) and finally eluted in 100 µl elution buffer.

**Table 2:** Sequences of primers and probes used in the study.

Name	Sequence 5′-3′	Genome position*	Reference
PRRSV-US-1dF	ATRATGRGCTGGCATTC	15257–15273	Kleiboeker et al., 2005, modified
PRRSV-US-1R	ACACGGTCGCCCTAATTG	15353–15370	
PRRSV-US-1TEX	ACACGGTCGCCCTAATTG	15307–15330	
PRRSV-HP-1F	CCGCGTAGAACTGTGACAAC	2914–2933	this study
PRRSV-HP-1R	TCCAGGATGCCCATGTTCTG	3035–3016	
PRRSV-HP-1Cy5aS	Cy5-ACGCACCAGGATGAGCCTCTGGAT-BHQ3	2941–2964	
PRRSV-EU-2.1F	GCACCACCTCACCCRRAC	14792–14809	Kleiboeker et al., 2005, modified
PRRSV-EU-2.1R	CAGTTCCTGCRCCYTGAT	14851–14868	
PRRSV-EU-2.1FAM	FAM-CCTCTGYYTGCAATCGATCCAGAC-BHQ1	14819–14842	
EGFP-11-F	CAGCCACAACGTCTATATCATG	537–558	Hoffmann et al., 2006
EGFP-10-R	CTTGTACAGCTCGTCCATGC	813–794	
EGFP-HEX	HEX-AGCACCCAGTCCGCCCTGAGCA-BHQ1	703–724	

* genome position according to.

type 1-PRRSV: type 1 prototype strain Lelystad (accession number M96262).

type 2-PRRSV: type 2 prototype strain VR-2332 (accession number U87392).

HP-PRRSV: Porcine respiratory and reproductive syndrome virus strain JXwn06.

(accession number EF641008).

EGFP: cloning vector pEGFP-1 (accession number U55761).

### Primers, Probes and Real-time PCR

Published sequence information of highly virulent, Chinese-type, isolates of PRRSV was used for the selection of primers and probes. Based on sequence alignments, primers and probes were selected with Beacon Designer 7.01 (PREMIER Biosoft International, Palo Alto, CA, USA). Primers and probes of the type 1 and 2 assays have been described previously [14]. In contrast to the type 2 assay by Kleiboeker et al. (2005), which uses two forward primers, a single modified forward primer was applied in this study. Furthermore the type 1 system was adapted using the available sequence information about European strains. Primers and probe of the EGFP internal control assay have been published by Hoffmann et al. [12]. Sequences of all primers and probes are shown in Table 2.

**Table 3:** Sequences of positive standards used in this study, primer and probe binding regions are depicted in capitals.

Name	Sequence 5′-3′
oLPC-PRRSV-NA1	gcc aat taa atc tca ccc cc
	**A CAC GGT CGC CCT AAT TG** a ata ggt gac tta gag gca caa
	**TGT CAA TCA GTG CCA TTC ACC ACA** cat tct aca ctg gca cga cag gtt tcc cga ctg gat gag atg cct caa
	**GAA TGC CAG CCC ATC AT** g ctg aga att ctc gcc cta tag tga gtc gta tta gaa ttc tga ttt att
oLPC-PRRSV-HP1	tct tgc ccc ccc gcc
	**TCC AGG ATG CCC ATG TTC TGC** gat ggt gct agg ggt att ccg tct gtg agg acg cag aca a
	**AT CCA GAG GCT CAT CCT GGT GCG T** ca tgg cac gac agg ttt ccc gac tgg agc gtt
	**GTT GTC ACA GTT CTA CGC GG** t gca att ctc gcc cta tag tga gtc gta tta gaa ttc tga ttt att
oLPC-PRRSV-EU1	aaa act gac ctt ccc gct gga tga aag cga
	**CGC AGT TCC TGC GCC TTG AT** t gaa agc
	**CGT CTG GAT CGA TTG CAA GCA GAG G** ga gct ggc acg aca ggt ttc ccg act gga gtt
	**CAG TCT GGG TGA GGT GGT GC** c aat tct cgc cct ata gtg agt cgt att aga att ctg att tat t

The multiplex PRRSV RT-qPCR was carried out using the RNA UltraSense™ One-Step Quantitative RT-PCR System (Invitrogen, Carlsbad, CA, USA). The multiplex assay was optimized using a total reaction volume of 25 µl. For a single reaction, 8.75 µl RNase-free water, 5.0 µl RNA UltraSense™ 5X Reaction Mix, 1.25 µl RNA UltraSense™ Enzyme Mix, 1.0 µl type 1-PRRSV-specific FAM-labelled primer-probe mix (20 pmol/µl PRRSV-EU-2.1F, 20 pmol/µl PRRSV-EU-2.1R, 6.25 pmol/µl PRRSV-EU-2.1FAM), 1.0 µl type 2-PRRSV-specific Texas Red-labelled primer-probe mix (10 pmol/µl PRRSV-US-1dF, 10 pmol/µl PRRSV-US-1R, 2.5 pmol/µl PRRSV-US-1TEX), 1.0 µl HP-PRRSV-specific Cy5-labelled primer-probe mix (10 pmol/µl PRRSV-HP-1F, 10 pmol/µl PRRSV-HP-1R, 2.5 pmol/µl PRRSV-HP-1Cy5aS) and 2 µl IC-specific HEX-labelled primer-probe mix (2.5 pmol/µl EGFP-11-F, 2.5 pmol/µl EGFP-10R, 1.25 pmol/µl EGFP-HEX) were combined in a master mix. Finally, 5 µl RNA template were added and the RT-qPCR was carried out in a Bio-Rad CFX 96 Real-Time Detection System (Bio-Rad, Hercules, CA, USA) using the following thermal profile: reverse transcription at 50°C for 15 min, initial PCR activation step at 95°C for 2 min; 45 cycles of denaturation at 95°C for 15 s, annealing at 56°C for 20 s, and extension at 72°C for 30 s. All samples were tested in duplicate.

**Figure 1:**
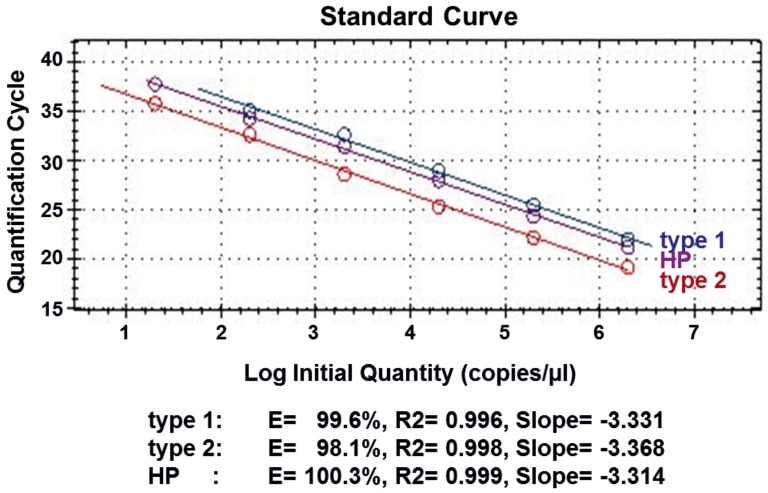
Analytical sensitivity of the PRRSV-multiplex-assay based on 10-fold dilution series of positive standard RNA. Regression curve was estimated by Bio-Rad CFX Manager™ Software (Bio-Rad, Hercules, CA, USA).

The commercially available ADIAVET® PRRS EU/NA Kit (ADIAGENE, Saint-Brieuc, France) for PRRSV detection and concurrent genotype differentiation was used according to the manufacturer’s recommendations.

**Figure 2:**
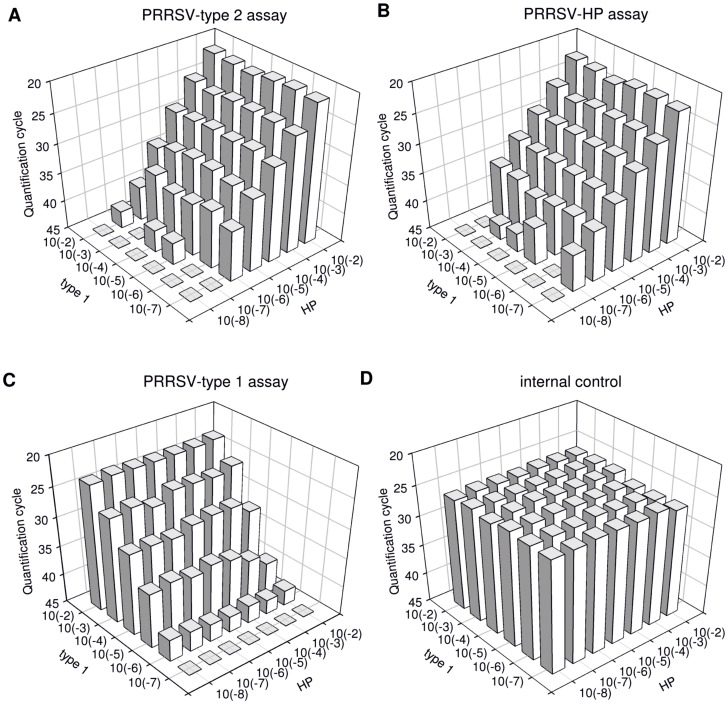
Chequerboard titration of a type 1- and a HP-PRRSV strain. Cq-values (z-axis) of the type 2- (2a) and HP- (2b) specific assays while co-amplification of different concentrations of the type 1 strain (y-axis) are shown. 2c depicts results of the type 1-specific assay when co-amplifying varying concentrations of HP-specific sequences (x-axis). Cq-values of the internal control RT-qPCR are shown in figure 2d. Images were designed supported by SigmaPlot® software.

### Positive Standard RNA

For each assay an oligonucleotide (oLPC-PRRSV-EU1, oLPC-PRRSV-NA1, oLPC-PRRSV-HP1) was designed containing a T7-promoter and the respective primer and probe binding sequences connected by short spacer sequences (Table 3).

The oligonucleotides were transcribed in vitro with the SP6/T7 Transcription Kit (Roche Diagnostics Deutschland GmBH, Mannheim, Germany) according to the manufacturer’s instructions. The T7-transcribed positive controls were digested with DNaseI and purified using the RNeasy Kit (Qiagen GmbH, Hilden, Germany). The RNA concentration was determined by spectrophotometry and the exact number of RNA molecules was calculated.

**Table 4:** Analyses of diagnostic sample from PRRSV infected swine with the multiplex RT-qPCR and with the ADIAVET® PRRS EU/NA Kit.

sample material	Multiplex PRRSV real-time RT-PCR				ADIAVET PRRS EU/NA		
	PRRSV type 2	HP-PRRSV	PRRSV type 1	IC	PRRSV type 2	PRRSV type 1	IC
	Cq	Cq	Cq	Cq	Cq	Cq	Cq
serum	>40	>40	33.31	26.35	>40	36.10	30.79
	>40	>40	35.87	27.02	>40	33.13	29.04
	>40	>40	24.89	24.82	>40	28.42	30.37
	>40	>40	24.45	28.14	>40	29.08	29.64
	>40	>40	23.06	28.32	>40	24.14	29.74
	>40	>40	22.49	27.27	>40	28.90	27.55
	>40	>40	29.78	26.85	>40	35.97	27.71
	>40	>40	24.21	27.87	>40	30.53	28.63
	>40	>40	29.55	26.21	>40	33.50	29.86
	>40	>40	29.75	25.60	>40	32.20	29.61
	>40	>40	29.36	25.65	>40	32.99	31.31
	>40	>40	32.63	28.15	>40	36.69	38.68
	34.79	>40	33.06	28.00	35.80	33.13	>40
	30.68	>40	>40	26.88	32.65	>40	36.64
	>40	>40	36.35	27.34	>40	31.44	>40
	>40	>40	32.52	28.92	>40	33.42	>40
lung	21.09	>40	>40	27.89	27.80	>40	28.03
	22.19	>40	>40	28.69	27.80	>40	22.93
	26.75	>40	>40	28.04	32.76	>40	25.68
	26.61	>40	30.35	28.52	34.28	30.46	25.20
	>40	>40	28.59	27.55	>40	28.48	24.56
	29.32	>40	>40	27.94	30.73	>40	24.35
	23.80	24.89	>40	26.10	>40	27.16	23.54
	29.49	30.15	>40	29.64	>40	34.25	22.71
	22.30	23.13	>40	>40	>40	25.89	22.98

### Animal Experiment with HP-PRRSV

On day 0 of the study, three serologically PRRSV negative swine (6–8 weeks of age) were inoculated intranasally with 10^5.8^ 50% tissue culture infective doses (TCID_50_) of the HP-PRRSV strain “China” in 10 ml. On day 3 post infection two further pigs were placed into the same room. During the entire study, blood samples were taken at regular intervals, rectal body temperatures were taken daily, and the pigs were monitored for clinical signs. On days 0, 3, 5, 7 and 10 after infection the quantity of white blood cells (WBC) was measured. Total RNA from serum was extracted and analysed with the multiplex PRRSV RT-qPCR. All animals were euthanized 14 days post infection. The animal experiment was reviewed by a state ethics commission and has been approved by the competent authority (State Office for Agriculture, Food Safety and Fisheries Mecklenburg-Vorpommern, ref. LALLF M-V/TSD/7221.3-2.1-024/08.

**Figure 3:**
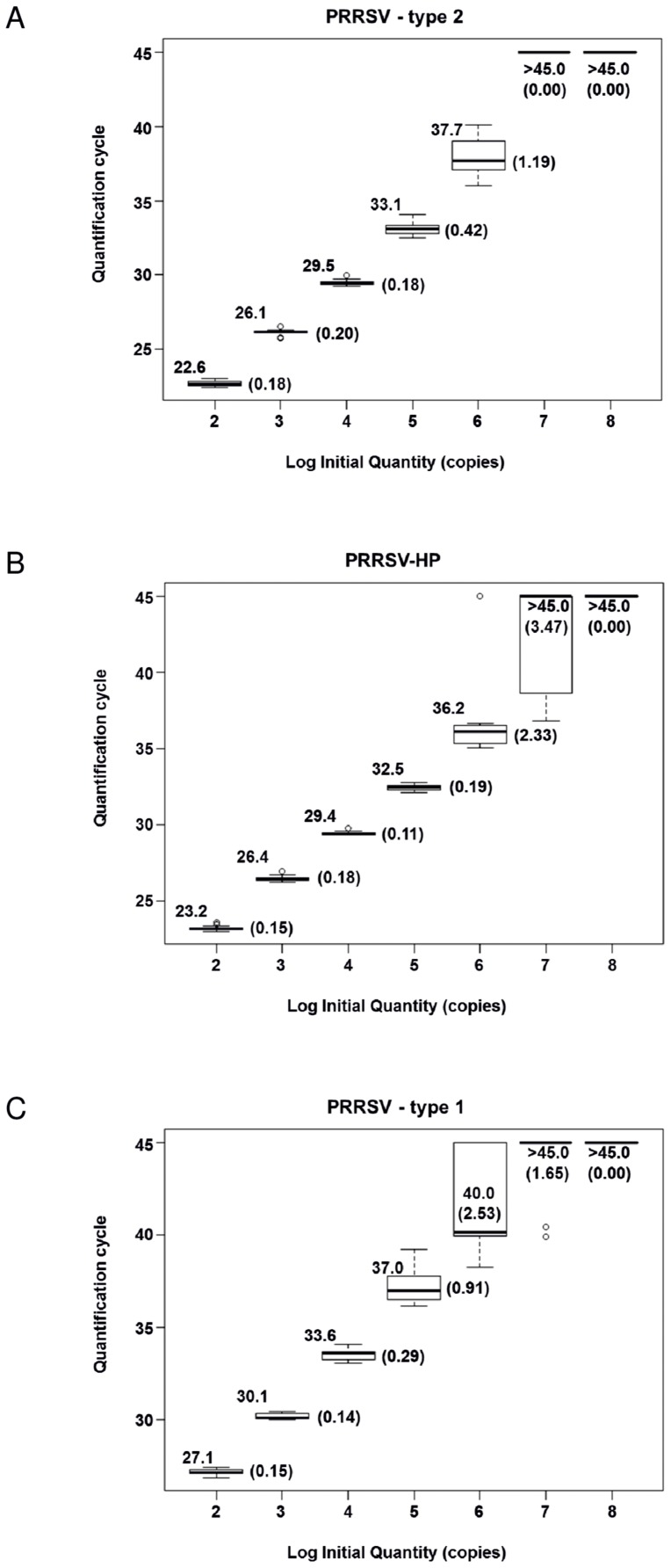
Reproducibility and repeatability of the multiplex PRRSV assay. The Cq-values (y-axis) of 16 replicates of the type 2- (3a), PRRSV-HP- (3b) and type 1- (3c) assays using 10-fold dilution series of adequate virus strains (x-axis: log 10 initial quantity) are shown. Mean values are indicated alongside of each box plot, standard deviations are depicted in parentheses. Box plots were designed supported by R software [38].

## Results

### Sensitivity

The analytical sensitivity of the multiplex PRRSV RT-qPCR was determined with a series of 10-fold dilutions of the positive standards. The type 2 and HP-assays showed an amplification efficiency of more than 98% with a linear dynamic range from 10^6^ copies down to 20 copies per µl (Fig. 1). For the type 1-assay, the linear dynamic range reached down to 200 copies per µl with a PCR efficiency similar to the type 2-assay (Fig. 1).

**Figure 4:**
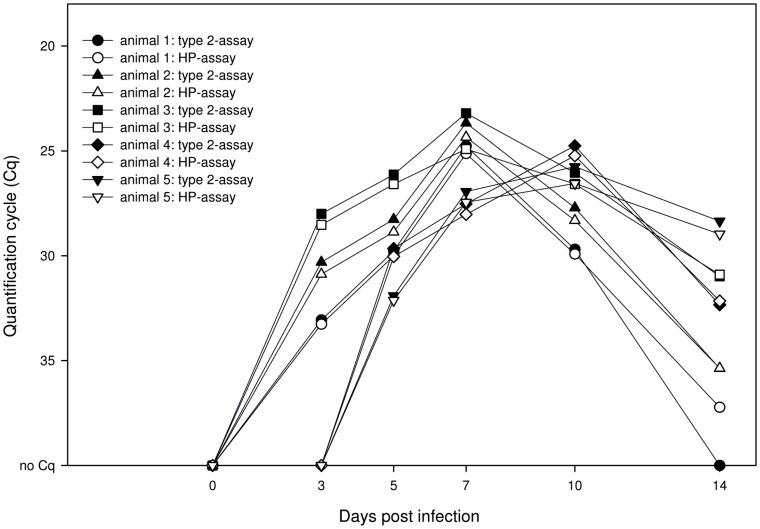
Analyses of serum samples from PRRSV-infected and in-contact pigs with the multiplex RT-qPCR. Graphs showing the same symbol belong to an identical animal, filled symbols depict results of the type 2-specific RT-qPCR unfilled of the HP assay. One to three are infected, four and five in-contact animals. Figure was designed supported by SigmaPlot® software.

The sensitivity of each PRRSV assay in the presence of the other genotype was tested in a checkerboard titration where the EU strain “Stendal V953” was titrated against the HP strain “China”. Internal control RNA was added to the RT-qPCR master mix. Since the HP-strains belong to type 2, amplification was observed in both RT-qPCR systems, PRRSV-HP and type 2-PRRSV. The sensitivity of the type 2 and HP-assay was hardly affected even by the co-amplification of high amounts of a type 1-PRRSV (Fig. 2a, 2b). Both assays showed comparable quantification cycle (Cq; [15]) values. The 10^−7^ dilution of the HP-strain scored positive in three samples out of six in the type2-assay, and four out of six in the HP system. In the samples which were tested negative in one or both assays, the concentration of the type 1-strain, also present in the reaction, ranged from 10^−2^ to 10^−7^. Independent of the HP- and type 2 strain amplification, type 1 PRRSV was reliably detected even in low concentrations (Fig. 2c). In addition, the IC-assay was obviously not affected by the co-amplification of PRRSV (Fig. 2d).

**Figure 5:**
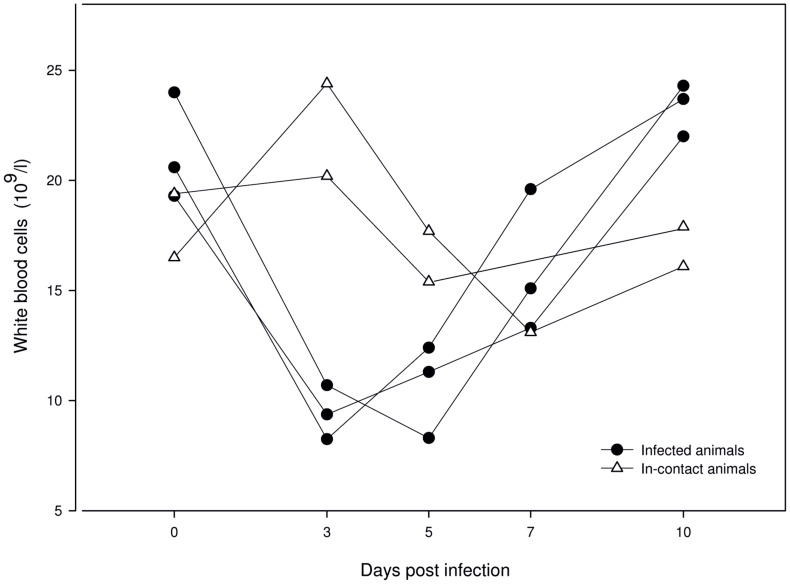
Quantity of white blood cells of PRRSV-infected and in-contact animals. Depiction was designed using the SigmaPlot® software.

The multiplex RT-qPCR was also compared to the ADIAVET® PRRS EU/NA Kit. Both systems achieved equal results for diverse virus strains (Table 1).

The diagnostic sensitivity was evaluated by testing different sample types, including serum and lung samples (Table 4). All samples showed comparable results when tested with the novel multiplex PRRSV RT-qPCR and the ADIAVET® PRRS EU/NA Kit. The multiplex PRRSV RT-qPCR assay failed to detect the IC in one case, the ADIAVET® PRRS EU/NA kit with two samples.

### Specificity

The sequences of primers and probes included in the PRRSV multiplex RT-qPCR were compared to published sequence information (NCBI GenBank) with a special focus on porcine viruses. There was no indication of possible cross-reactions.

The diagnostic specificity of the multiplex RT-qPCR assay was validated using 132 serum samples from animals previously tested negative for PRRSV by a commercial RT-qPCR kit. All scored clearly negative in the type 1 and 2, as well as PRRSV-HP-specific assay within the multiplex approach with positive results in the IC-specific RT-PCR.

When testing the virus strains (Table 1) as well as the diagnostic samples (Table 4), the multiplex PRRSV RT-qPCR and ADIAVET® PRRS EU/NA Kit correctly determined the genotype for all samples.

### Reproducibility, Repeatability

Intra-assay reproducibility of the developed multiplex real-time RT-PCR was assessed with four replicates each of a 10-fold dilution series of RNA from PRRSV strains “USA VD 28775/2″, “Stendal V953” and “China”, respectively.

The inter-assay repeatability was determined for 10-fold dilution series on four separate days. Mean values and standard deviations of the PRRSV specific assays are shown in Fig. 3. The IC-specific PCR showed a mean Cq value of 33.86 with a standard deviation of 0.72.

### Animal Experiment with HP-PRRSV

The body temperature of the infected pigs reached 40°C at day 3 post infection (dpi) and 41–42°C at 8 dpi. The fever then lasted until the end of the study. In-contact animals reached 40°C 3 days after first exposure and more than 41°C after 8 days. Monitoring of clinical signs revealed symptoms like red to purple dermal discoloration and purple and blue ears 5 days after infection or 7 days after first exposure, respectively. The discolorations persisted until the end of the study. Additionally, one of the infected and one of the in-contact animals showed diarrhoea and nasal discharge. Coughing was observed in one of the infected animals, while sneezing was seen in one of the in-contact animals.

Serum samples of infected and in-contact animals were investigated using the multiplex PRRSV RT-qPCR (Fig. 4). Samples from day 0 of the study scored negative for all animals. On 3 dpi the infected animals showed positive results, while the in-contact pigs remained negative. On 5 dpi PRRSV-RNA could be detected in samples of all animals.

In serum samples of the infected animals, Cq values declined until 7 dpi, and subsequently increased till 14 dpi. The in-contact animals showed decreasing Cq values up to day 10, followed by increasing values also until the end of the study. All samples showed equal results in the type 2 as well as the HP-specific system (Figure 4). However, one weak positive sample scored negative with the type 2-specific assay, whereas the HP-specific assay gave a positive result.

The WBC count of infected animals dropped from 0 dpi until 3 dpi, and subsequently increased again. In-contact animals showed a decreasing number of WBC from day 3 until day 7 (Figure 5).

## Discussion

In 2006, a highly pathogenic disease emerged in swine farms in China. The morbidity rate was 50–100% and the mortality rate was 20–100% [16]. In the affected swine herds, high and continuous fever, anorexia, red discolorations in the bodies and blue ears were observed, followed by diarrhoea and other clinical signs in the late phase of the disease. A highly pathogenic PRRSV type with a 30-aa deletion within the Nsp2 encoding genome region could be demonstrated as the causative agent. Under experimental conditions, the virus could induce clinical symptoms identical to those observed in the field [7], [17]–[19]. However, none of these trials used in-contact control animals to study virus transmission.

In our animal experiment the typical clinical symptoms of high and continuous fever and dermal discolorations could be observed in infected as well as in contact animals. The in-contact animals showed clinical symptoms 3 days later than the infected swine. As the control animals were placed in contact to the infected swine on day 3 of the study, a very rapid infection has to be assumed. This is reinforced by the decrease of WBC two days after the first exposure. Samples from the animal trial were used to validate the newly developed PRRSV multiplex assays, allowing a differentiation of type 1 and 2 as well as a direct characterization of a PRRSV-strain as the HP-type. This is possible, since the Nsp2-encoding region within the 15 kb genome of PRRSV [20] shows a remarkable genetic variation [21], [22]. Although the 30-aa deletion in Nsp-2 coding region of HP-PRRSV is not related to its virulence [23], the deletion can be used as a marker to distinguish the highly pathogenic Chinese virus from other NA-PRRSV strains.

For the detection of HP-PRRSV several RT-qPCR assays have been developed [8], [11]. None of these assays, however, can detect other type 2- or even type 1-strains. Furthermore, RT-PCR assays for the detection and differentiation of both genotypes have been described [14], [24], [25], but no HP-PRRSV detection system was included in these assays.

Type 1 PRRSV isolates have been recently introduced into North American swine herds infected with type 2 strains [2]. Natural dual infection of swine with strains of both genotypes has been documented in Europe [26]. Even in two diagnostic samples used in this study, the type 1 and 2specific assays scored positive, emphasizing the necessity for simultaneous detection and differentiation of both genotype strains.

By testing dilution series of appropriate positive standards, it was demonstrated that the novel multiplex PRRSV RT-qPCR system is a highly sensitive approach for the detection and differentiation of type 1 and 2 PRRSV and allows also the direct characterization of HP-specific genome sequences. Checkerboard titration confirmed that the sensitivity of the respective assays was not affected by the co-amplification of any other target included in the multiplex assay. In the lowest detectable dilution of HP-PRRSV either the HP- or the type 2 assay or both scored positive, independently of the concentration of the type 1-strain RNA in the same sample. Consequently, the presence of HP-PRRSV genome in weak type 2-positive, and HP-negative, samples cannot be fully excluded.

When testing diverse virus strains and diagnostic samples including serum and lung samples, the new multiplex RT-qPCR showed a high diagnostic sensitivity. However, the number of used strains is limited consequently not representing the full range of the diversity of both genotypes. Especially the Eastern European type 1 isolates are highly diverse [27], [28]; one of those strains (“Lena”) was tested by the multiplex RT-qPCR and correctly detected. Sequence information of Eastern Europe isolates, in particular ORF 7 sequences, which covers the primers and probes used in this study, are scarce. As a consequence, the applicability of the multiplex PCR for those strains in not completely proven. Nevertheless, the known sequences of primers and probes enable a rapid adaption to new sequence information and emerging strains, which is an important feature in PCR diagnostics of viruses exhibiting a large genetic diversity, such as PRRSV [29]–[32].

In addition to the PRRSV type 1, 2 and HP-specific assays, an internal control PCR based on heterologous RNA sequences was introduced. To verify efficient RNA extraction and uninhibited amplification, IC based on housekeeping genes [33]–[36] or heterologous internal controls [12], [37] are used. Depending on the quality of the clinical sample, even housekeeping genes have unknown and variable mRNA concentrations, which could negatively influence the amplification and detection of the virus-derived nucleic acid. As the concentration of the heterologous IC RNA is known, sub-optimal RNA extractions or partial inhibitions of the RT-PCR can be detected, therefore avoiding false-negative results. However, a negative result in the IC specific PCR could also be caused by the absence of detectable RNA, but this is not significant when the virus-specific PCR scored positive.

A large number of samples from PRRSV-negative pigs were also tested and scored clearly negative in the PRRSV assays, whereas all of them scored positive in the IC-specific RT-qPCR. Using the checkerboard titration, it could be shown that the IC-specific assay was not affected by the co-amplification of PRRSV.

In serum samples obtained from the animal trial, viral genome was first detectable at day 3 after infection or first exposure, demonstrating the rapid infection of the introduced contact animals. Except in a weak positive sample, where the HP-assay scored positive and the type 2 negative, both detection systems showed comparable Cq-values.

In conclusion, the newly developed 4-colour multiplex RT-qPCR is a sensitive and specific method to confirm the presence of PRRSV genomes in field samples. For the first time, this assay can also detect and distinguish type 1 and 2 PRRSV as well as the Chinese HP-strain in a single qPCR reaction.
